# The American Dental Club of Paris

**Published:** 1898-02

**Authors:** 


### Obituary.

### THE AMERICAN DENTAL CLUB OF PARIS.

### RESOLUTIONS PASSED AT SPECIAL MEETING OF DECEMBER 11, 1897.

At a special meeting of the American Dental Club of Paris, held at the office of its president, Dr. G. C. Daboll, on Saturday evening, December 11, 1897, the following resolutions were unanimously adopted:

Whereas, By the sudden death of Thomas W. Evans, M.D., D.D.S., Ph.D., which occurred at his home in Paris, Sunday evening, November 14,

1897, this Club loses one of its most assiduous members, and our profession one of the most remarkable men that has ever graced its ranks; therefore be it

*Resolved*, That the American Dental Club of Paris deeply deplores the death of so eminent a colleague, who, as its first president and as a fellow-member, ever alert to the interests of the Club and the profession, always commanded the profound respect of all.

*Resolved*, That we as a body of American dentists whose lot by various circumstances has fallen in a foreign land, while gratefully acknowledging the hospitality of our sister republic, and our gracious adoption by her people, feel it but just to acknowledge our gratitude to Dr. Evans, who, as one of the great pioneers of the dental profession, has done so much to break down old prejudices and prepare the way not only for us, but for every dentist whose heart is in his work, and whose object is to benefit mankind.

*Resolved*, That we regard his success in securing the recognition, by all the nations of Europe, of the beneficence of dental science and art as first understood and practised in America, as of the greatest importance to the public, as well as to the dental profession.

That influence was strongest during the first twenty-five years of his practice, during the plastic period of the evolution of dental science so especially active in America.

On account of his influence in the highest circles, the way has been made easier to convert conservative Europe to modern methods of conservative dentistry, and not only every member of this Club and every American dentist of Europe, but every native dentist as well, has been benefited by that influence; and we believe, above all, by the adoption of modern methods, such a stimulus has been given to *all* dentists of *all nationalities* as will one day render dental science a *universal* and not merely a national science, as it was during a great portion of Dr. Evans's career.

*Resolved*, That while we recognize the influence of others of his contemporaries, he played a principal *rôle*, owing to the peculiar position brought by his unparalleled success, such success being due to his personal magnetism, high-mindedness, affability, practical common sense, and tact.

*Resolved*, That this Club regard the numberless honors conferred upon Dr. Evans by the various sovereigns of Europe, as the just tokens of appreciation of the dental profession through one of its great representatives, and it is proud that he was an American, and proud he was a member of this Club.

And, notwithstanding his pecuniary success, his unlimited honors, and his long sojourn away from his native land, we know that while being faithful to his duties in foreign lands his loyalty and affection for his own country never diminished.

He was first, last, and always a dentist, and proud to be considered one, and despised that *snobism* which makes some men ashamed of the profession to which they owe all their success in life.

*Resolved*, That we believe the name of Dr. Thomas W. Evans deserves a place with other great names in the history of the development of dental science.

*Resolved*, That our sympathy be extended to the relatives and friends of Dr. Evans; that a copy of these resolutions be handed them; that a full record be made and preserved by the secretary of the Club in its procedures; and that a copy be sent to the dental journals of America for publication.

*Resolved*, That as a token of respect to our late *confrère*, the American Dental Club of Paris join in a body to attend his funeral.

JOHN W. CRANE,
ISAAC B. DAVENPORT,
J. H. SPAULDING,
*Committee.*

